# Design, implementation, and evaluation of Students as Partners interactive feedback model

**DOI:** 10.1152/advan.00182.2022

**Published:** 2023-01-12

**Authors:** Nicole A. Setterington, Sarah McLean, Anita Woods

**Affiliations:** ^1^Department of Anatomy and Cell Biology, Western University, London, Ontario, Canada; ^2^Department of Physiology and Pharmacology, Schulich School of Medicine and Dentistry, Western University, London, Ontario, Canada

**Keywords:** action research, COVID-19, feedback, student partnerships, teaching and learning

## Abstract

In September of 2020, a group of dental students (DDS) and motivated faculty at the University of Western Ontario came together in response to the pandemic and established a real-time feedback model. The goal of this model was to address technical challenges following the quick transition from in-person courses to a fully online format for student learning. This initial offering formed the foundation of the Students as Partners (SaP) program to identify and address technical and curricular issues. We used an action research approach to evaluate and refine the innovation’s delivery. Preliminary data from the first cycle suggested that students were unaware of the impact of their feedback and the actionable items from their feedback. Thus, for the second iteration we focused on making the entire process more transparent by using Padlet as a way to streamline posting and responding to feedback. To evaluate the refined system, we distributed surveys to student and faculty participants to obtain feedback on their awareness and satisfaction and effectiveness of the program. For students who utilized the system, the majority indicated that they were informed of changes based on their feedback. Furthermore, students reported that our innovation provided a platform for the student voice. Faculty impressions were generally positive, and the majority of faculty respondents indicated that they implemented changes to their content/curriculum based on feedback. These results demonstrate that the SaP program’s real-time feedback system closed the feedback loop and facilitated real-time improvements based on actionable feedback. To our knowledge, this is the first study to design, implement, and evaluate a real-time feedback system for the purpose of modifying how an instructor teaches.

**NEW & NOTEWORTHY** Course feedback surveys at the end of term infrequently result in beneficial change. However, student feedback should be considered to develop meaningful learning. In response to this problem, we report on a novel Students as Partners innovation to address instructional issues in real time with a virtual bulletin board application embedded in the learning management system. Students and instructors valued the system’s ability to close the feedback loop and provide transparent, actionable change.

## INTRODUCTION

As higher education institutions transitioned to fully online teaching and learning environments because of the COVID-19 pandemic, the importance of effective pedagogical models was reinforced. This abrupt change of curriculum delivery highlighted weakness in the transition to online course delivery but also amplified the importance of efficient communication between learners and educators.

The opportunity to obtain student feedback can be a useful tool for guiding changes in pedagogical design. The traditional end-of-course evaluations are common models currently employed in many higher education institutions and often provide the only opportunity for student feedback (1). Although student feedback at the end of term at our institution and others has been routinely collected, it fails to provide change while courses are occurring. Furthermore, it has been demonstrated that these types of instructor evaluations are biased and therefore, when used as a measure of teaching effectiveness, are problematic (2, 3).

One approach that has been frequently used to drive educational innovations in higher education is Students as Partners (SaP). SaP is an inclusive movement that embraces students and faculty working together on teaching and learning (4). The program offers students the ability to play active roles in their learning process by offering valuable feedback and allowing them to contribute to shaping teaching and learning. SaP encourages academic collaborations and recognizes the reciprocal partnerships developed from different, but useful, forms of expertise offered by faculty and students (5).

An essential element, the student voice, provides an insider perspective of the student’s learning (6–8). By integrating student feedback in curriculum content, teaching delivery models, and assessment models, students can be much more than the passive receiver or consumer of their education (6, 9). Opportunities that allow for such roles can help build a sense of purpose and engagement for invested students’ future careers. However, if feedback is not implemented or the action of the feedback is not clearly communicated, the students who provided it may be less likely to contribute further and may disengage in their learning (10).

In end-of-course evaluations, there can be a lack of accountability for faculty to make changes, even if there may be good evidence for modifications (11). Students are usually unaware of the outcomes of their comments and whether instructors implemented changes for future iterations of the course. Thus, the feedback from students is unresolved, and the feedback loop is not closed (12). SaP programs have an opportunity to address the failure to close the feedback loop seen in other models of course evaluations. When collaborative partnerships built on the ethic of reciprocity are established, and when attempts are made to alert students of the value of their contributions, the prospect of students continuing to provide feedback in the future will subsequently improve (10). Therefore, emphasis on closing the feedback loop is a critical component of the design of a SaP program for curricular innovation.

To create effective engagement in quality improvement practices, the perceptions of students need to be integrated into a four-step cycle (10). This cycle consists of listening, critical analysis, implementation, and feedback. Although it has been recognized that not all suggestions provided by students will always be applicable, appropriate, or constructive (13), what is likely most important is the follow-up response on why some of their suggestions could not be implemented. Executing this addresses the four-step cycle for quality improvement practices with the emphasis on closing the feedback loop (10).

To evaluate the impact of our system in promoting pedagogical change, we first sought to determine whether our SaP model supports students’ reporting of technical issues. To investigate this, we developed an interactive feedback model. It was recognized that in addition to our dentistry student population that was evaluated in our first iteration, similar challenges for online teaching and learning existed in undergraduate education programs. Therefore, in our second iteration we extended the SaP program to a subgroup of third-year physiology courses within the undergraduate Bachelor of Medical Science degree (BMSc) program.

Here we present two iterations of design to our interactive feedback system using an action research approach. We sought to determine whether our design resulted in utility for both students and faculty and whether it was effective in closing the feedback loop. Furthermore, we investigated whether there were any differences of perceptions of the feedback system between our two populations of students who participated in the study, based on the types of feedback provided and responses by users of the system.

## MATERIALS AND METHODS

### Study Design and Population

This study was conducted at the University of Western Ontario Schulich School of Medicine and Dentistry. In response to the motivated group of dentistry students and faculty who initiated an online feedback model to address technical issues in course delivery in September of 2020, we assisted to formalize the SaP program. All students enrolled in the Doctor of Dental Surgery (DDS) program, including internationally trained dentists, were invited to participate in the first cycle of the program. For the second cycle, DDS students and undergraduate students in the Bachelor of Medical Science (BMSc) program who were enrolled in three program-required undergraduate courses, advanced human physiology, cellular physiology, and a laboratory in physiology and pharmacology, were invited to participate.

This study employed an action research approach in which the study design followed a cyclical process (14). As prescribed by action research, we developed a plan for improvement, implemented the plan, and observed and documented the implementation, followed by reflection on the effects and planning for informed action for subsequent iterations.

### Ethical Approval

This study was reviewed by the Western Research Ethics Manager Non-Medical Research Ethics Board (NMREB). It was determined to meet the criteria for institutional research and was approved (Project ID No. 118802). After participants read the letter of information, consent was implied by clicking an explicit box to access the survey for the study.

### Measures

Our study evaluated two cyclical processes of action research. *Cycle 1* of our research examined the efficacy of a SaP program in a group of first- and second-year dentistry students in the 2020–2021 winter term. Based on data from *cycle 1*, *cycle 2* redeveloped and examined a real-time feedback system that focused on closing the feedback loop and expanded to the population of undergraduate BMSc students in the 2021–2022 winter term.

### Cycle 1

#### Cycle 1 feedback system setup.

**Figure 1.**
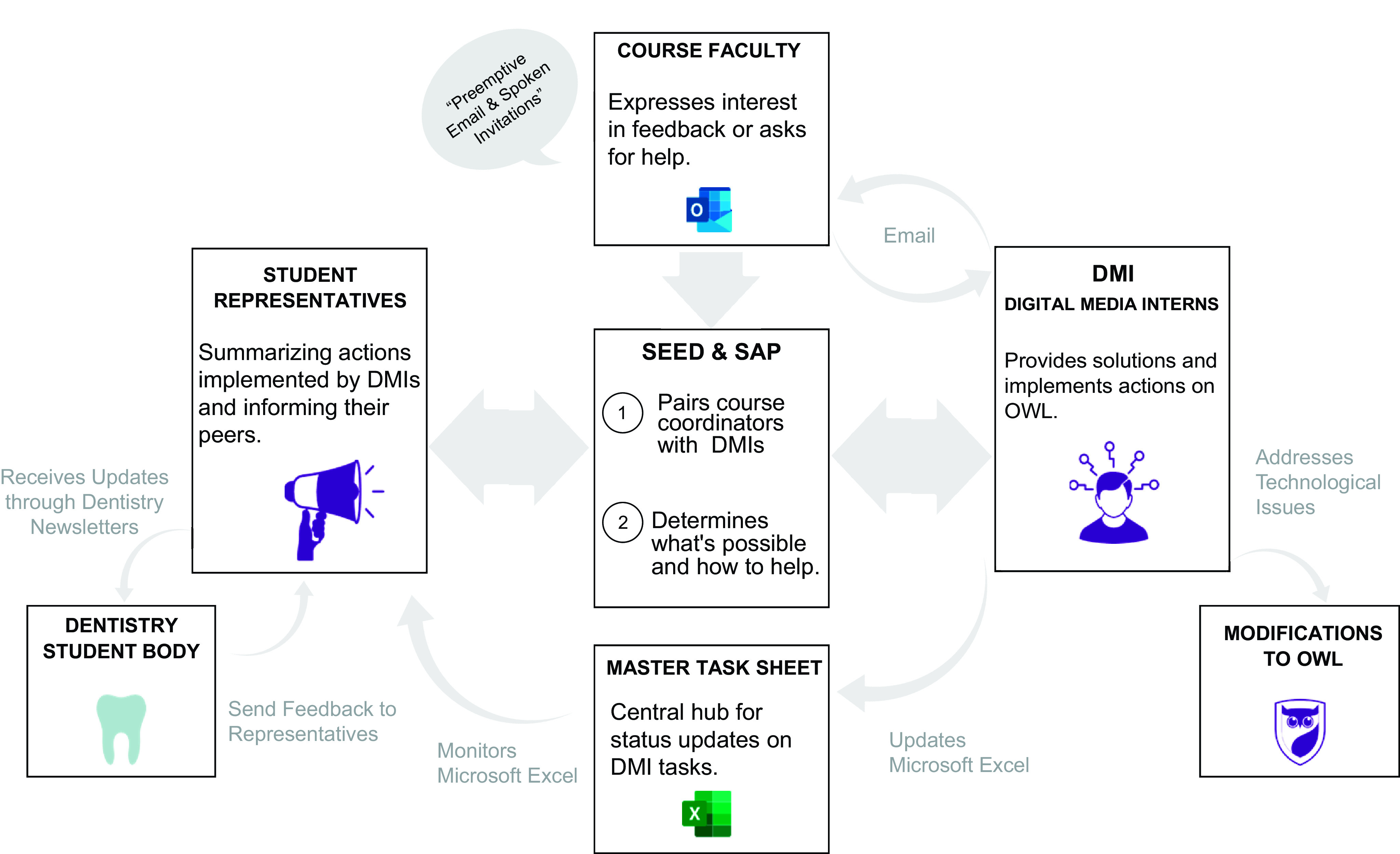
A schematic of the feedback system designed for *cycle 1*. Beginning with the dentistry students, feedback was collected and anonymized by student representatives, providing this to the Schulich Education Enhancement Division (SEED) by a master task Excel spreadsheet. This division connected course coordinators with digital media interns (DMIs), who were also Students as Partners (SaP) to provide solutions by correcting issues on the learning management system, OWL, and/or communicating with faculty involved in the courses. When feedback was able to be addressed, dentistry student representatives provided newsletters containing the information back to the dentistry student body.

The *cycle 1* feedback system (Fig. 1) was developed in collaboration with faculty and elected DDS class presidents. The initial idea for the feedback system was to automate the process as much as possible with Microsoft Power Automate. Elected DDS class presidents in this iteration acted as the primary SaP student representatives. Essentially, if a DDS student had a technical or other issue in the course, they would inform their class president, who would keep track of the feedback on an Excel spreadsheet and then submit a ticket with Power Automate to the Schulich Education Enhancement Division (SEED), a centralized technology support unit. The Director of SEED would then determine whether the issue was actionable and, if actionable, would assign a digital media intern (DMI) to address the issue through the learning management system (LMS). DMIs were current students with specialized training in the LMS as well as additional technical skills, who worked in a SaP relationship with faculty and SEED. The DMI would then contact the faculty member for the course and resolve the issue. The DMI would also inform the class president about whether the issue was handled, which the class presidents communicated to the student body via newsletters.

*Cycle 1* involved DDS students in their first or second academic year, as well as faculty who instructed courses for these cohorts. After implementing the SaP program and feedback system framework, we observed and reflected on the impact of this system on the overall teaching and learning experience (Fig. 1). Data were collected during this cycle to refine the next iteration of the program, which we have termed *cycle 2*.

#### Cycle 1 measures.

At the end of the winter 2021 term, surveys generated in Qualtrics (Qualtrics, Provo, UT) were distributed to obtain student and faculty perspectives of the awareness, use, and effectiveness of the SaP program feedback system. More specifically, these surveys helped assess whether the feedback system was successful in meeting the requirements of a true SaP program, including closing the feedback loop, and whether there was evidence of an enhancement of teaching and learning.

Surveys were distributed to two populations: *1*) DDS students in second and first years of studies and *2*) all dentistry core faculty who opted in to the SaP program. These surveys included seven-point Likert scale and open-ended response questions. These metrics allowed us to obtain the percentage of respondents who overall agreed or disagreed with our statements regarding the feedback system and the provision of comments regarding the program.

#### Cycle 1 participants.

Of the 149 DDS students who were invited to participate in the study by answering the survey, 15% consented (*n* = 22). Not all those who consented responded to all statements in the survey: for awareness statements *n* = 11, for use-satisfaction statements, those who responded “yes” to using the feedback system *n* = 4 or 5, and for effectiveness statements *n* = 9 or 10.

Twenty-eight DDS faculty were invited to join the SaP program. Fourteen DDS faculty opted in and received a separate faculty survey. Of the 14 faculty members who received the survey, 57% (*n* = 8) consented and responded to the survey.

### Cycle 2

#### Cycle 2 feedback system setup.

After reflecting on the preliminary data from *cycle 1*, we focused our research question and redesigned the feedback system to better serve students and faculty. To better investigate a feedback system that supports student feedback, we modified the technological framework of the SaP model.

The focus in the second cycle was to more efficiently close the feedback loop in real time. To ensure anonymity in the feedback system, a digital bulletin board application was selected, called Padlet (Padlet, San Francisco, CA). Padlet is an interactive web platform that provides an easy way to create and collaborate. By embedding Padlet into the institution’s LMS, the revised model allowed students to efficiently provide ongoing course feedback and report issues related to content delivery.

**Figure 2.**
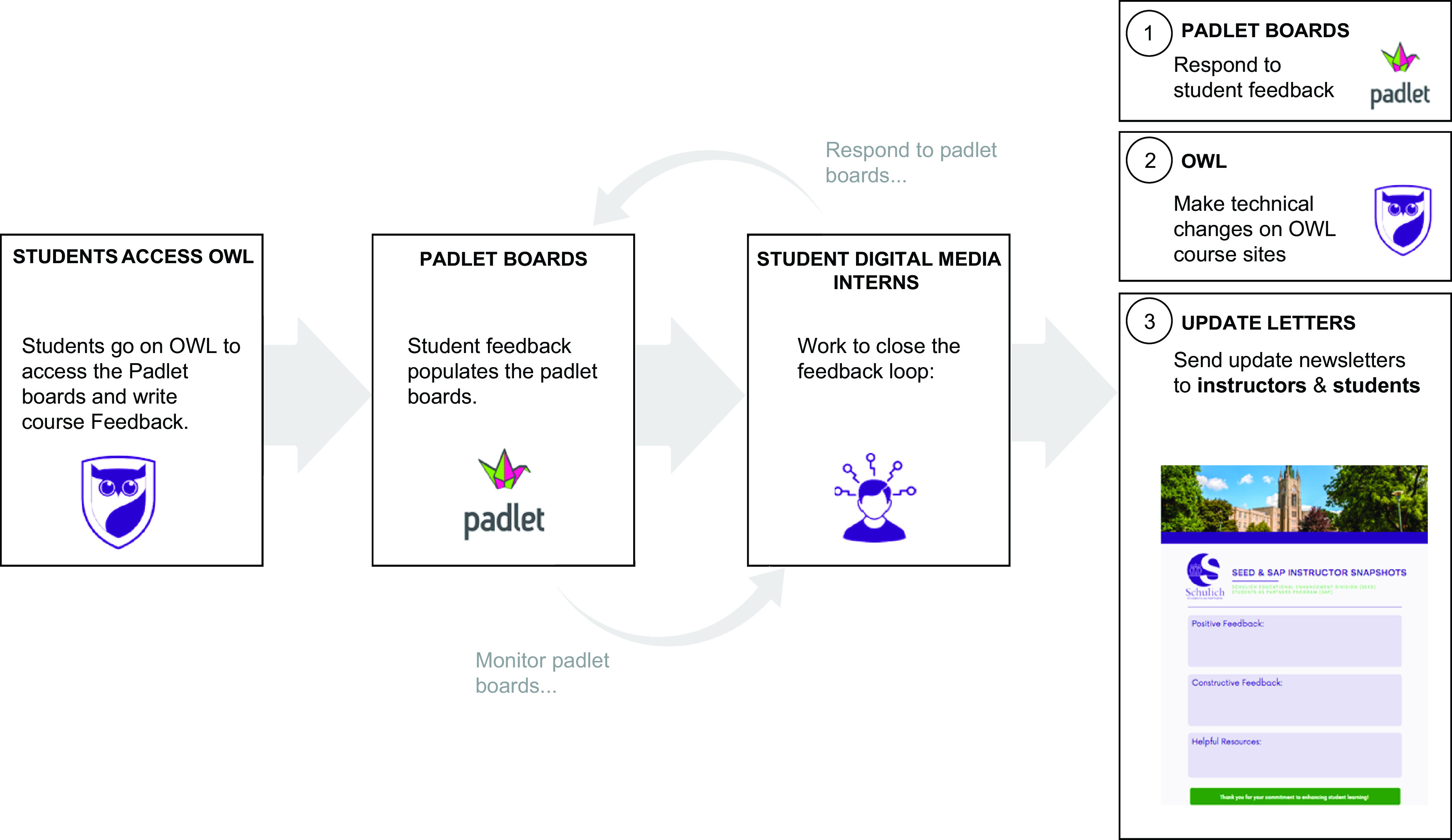
*Cycle 2* feedback system design. This feedback system schematic displays how students could efficiently provide ongoing anonymized course feedback and report issues related to content delivery directly to student digital media interns. Updates to feedback were posted directly back to the comments posted on the Padlet boards. Padlet is a collaborative platform technology that was embedded into Western’s learning management system, OWL.

In addition to maintaining the anonymity of students, Padlet recorded the time stamps of the feedback. This transparency allowed students to report and see any feedback that was previously posted, they could “upvote” or “downvote” the feedback, and they could also directly respond to the feedback on the same post, providing some context to the number of students with the same concerns/issues (Fig. 2). SEED representatives monitored the class Padlets, and when an actionable issue arose a DMI was assigned to deal with the issue and also inform the faculty member, similar to *cycle 1*. Importantly, SEED representatives were able to comment directly on the Padlet and respond to individual queries, which removed the need for newsletters. Furthermore, this allowed all dental students the opportunity to post feedback, without it being filtered through the class presidents. In this way, *cycle 2* of our feedback system engaged all students, rather than simply the class presidents.

The population that had access to the SaP feedback system was expanded to include three large third-year physiology and pharmacology courses offered to undergraduate students in the BMSc program. Thus, *cycle 2* involved DDS students in first, second, and third years of their program and undergraduate BMSc students who were in their third and fourth years of studies, as well as the faculty members who managed the relevant courses in these programs.

#### Cycle 2 measures.

To evaluate *cycle 2* at the end of the fall 2021 term, surveys generated in Qualtrics were distributed to obtain student and faculty perspectives. Some questions were modified and refined from the *cycle 1* survey but still focused on the awareness, satisfaction, and effectiveness of the SaP program feedback system. These surveys were distributed to three populations: *1*) DDS students in first, second, and third years; *2*) BMSc students enrolled in three selected third-year physiology and pharmacology courses; and *3*) all dentistry and BMSc course directors who opted in to the SaP program.

Similar to *cycle 1*, these surveys included seven-point Likert scale and open-ended response questions, allowing us to evaluate the percentage of respondents who overall agreed or disagreed with our statements regarding the feedback system.

#### Cycle 2 participants.

Overall, 227 DDS and 549 BMSc students received the survey. Of the 227 DDS students who received the survey, 20% consented (*n* = 45). Not all respondents answered all questions: for answers to awareness statements *n* = 36–38, for satisfaction statements those who responded “yes” to using the feedback system *n* = 8 or 9, and for effectiveness statements *n* = 28 or 29. Of the 549 BMSc students who received the survey, 24% consented (*n* = 131). Not all respondents answered all questions: for answers to awareness statements *n* = 121, for satisfaction statements those who responded “yes” to using the feedback system *n* = 36 or 37, and for effectiveness statements *n* = 79–92. Of the 14 faculty members who opted in to the SaP program and received the survey, 87% consented (*n* = 12). Not all respondents answered all questions: for awareness statements *n* = 10, and for feedback implementation statements *n* = 6–9.

### Qualitative Analysis

We had two sources of qualitative data for *cycle 2*. We had open-ended questions through the surveys that were subjected to analysis. Second, we evaluated the Padlet posts to examine the type of feedback/issues that students were reporting through the SaP system. We analyzed each feedback item posted on each course’s Padlet for common themes.

Qualitative data from *cycle 2* were analyzed with ATLAS.ti 9 software (Scientific Software Development GmbH, Berlin, 2020). The data were analyzed with the six-step method as outlined by Braun and Clarke (26). The emerging themes were developed concurrently as the data were analyzed by one coder (N.A.S.). Codes from the same semantic domains were applied in a mutually exclusive manner. This method was used to develop a codebook. After the initial analysis, the codes attributed to the textual data were removed, and a second coder (A.W.) analyzed the data using the codebook developed by N.A.S. The main themes identified by the two independent coders were the same, although the number of attributable quotes differed slightly.

### Statistical Analysis

To quantify the Likert statements, numerical values were assigned to the corresponding agreement. Respondents to all surveys chose from a seven-point scale (from “strongly disagree” to “strongly agree”) to indicate their level of agreement with statements regarding the SaP program. All data presented summarize the average Likert response by respondents in agreement with a statement (i.e., who answered either “somewhat agree,” “agree,” or “strongly agree”) (15). “Strongly disagree” was assigned the numerical value 1, “disagree” = 2, “somewhat disagree” = 3, “neutral” = 4, “somewhat agree” = 5, “agree” = 6, and “strongly agree” = 7. For the data sets that were posed in the negative, scale data were reverse coded, from 7 being substituted for “strongly disagree” to 1 for “strongly agree.”

These data from *cycle 2* were analyzed with GraphPad Software Prism version 9.2.0 for Mac (San Diego, California, 2021) after exporting data from Qualtrics and Microsoft Excel (version 16.0, Microsoft Corporation, Redmond, WA).

Ordinal data comparing Likert scale scores about SaP program awareness, satisfaction, and effectiveness of DDS and BMSc students were examined for relationships with a two-way ANOVA mixed-effects test. After the mixed-effects analysis a Šídák’s multiple-comparisons test (post hoc) was performed to identify which specific means were significant from the others, and a *P* value of <0.05 was considered to be significant.

## RESULTS

### *Cycle 1*: Awareness, Use-Satisfaction, and Effectiveness

**Figure 3.**
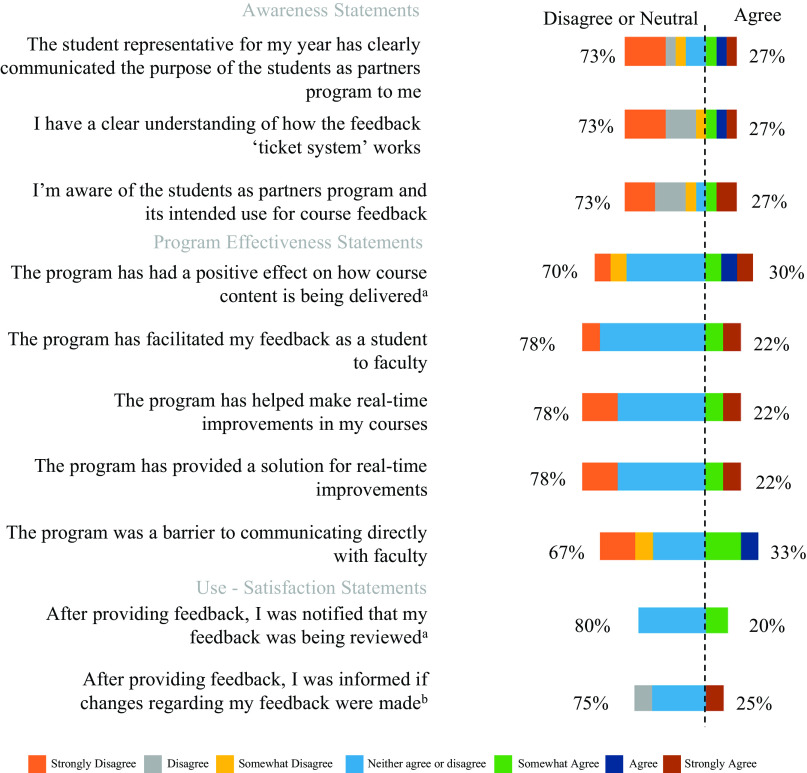
*Cycle 1* of the Students as Partners (SaP) program. Percentage of DDS students’ agreement with statements regarding their awareness and use-satisfaction and the effectiveness of the SaP program. The colors represent the 7 different levels of agreement, ranging from “strongly disagree” to “strongly agree.” The length of the colors represents the percentage of students within each agreement category. The dashed line indicates the point between “neutral” and “somewhat agree” (awareness *n* = 11; effectiveness ^a^*n* = 10, otherwise *n* = 9; use-satisfaction ^a^*n* = 4 and ^b^*n* = 5).

In the first iteration of our innovation (*cycle 1*), DDS students and participating faculty members were surveyed. Both surveys assessed awareness, satisfaction, and effectiveness of the SaP feedback program and solicited comments on program strengths and weaknesses (Fig. 3). Only 27% of student respondents agreed that they were aware of the SaP program (*n* = 11). Furthermore, 22% agreed that the program facilitated their feedback to faculty or made real improvements in their courses (*n* = 9). Not surprisingly, only 30% agreed that the program had a positive effect on how course content was delivered (*n* = 10). Finally, low levels of satisfaction on how and whether their feedback was implemented were reported, with only 20% of student respondents agreeing that they were notified when their feedback was being reviewed (*n* = 5) and 25% agreeing that they were informed if changes were implemented by faculty (*n* = 4) (Fig. 3).

The response rate from core faculty in the survey was minimal (*n* = 3 and 4), but it pointed out areas for improvement to our feedback system that were similar to the themes observed from the student respondents (data not shown).

*Cycle 1* clearly identified a need to improve the awareness of the program, but, more importantly, it identified that students need to be made aware of when changes are implemented or when their suggestions are not possible. We therefore focused on closing the feedback loop by making the responses to student suggestions as transparent as possible.

### *Cycle 2*: Awareness, Use-Satisfaction, and Effectiveness

**Figure 4.**
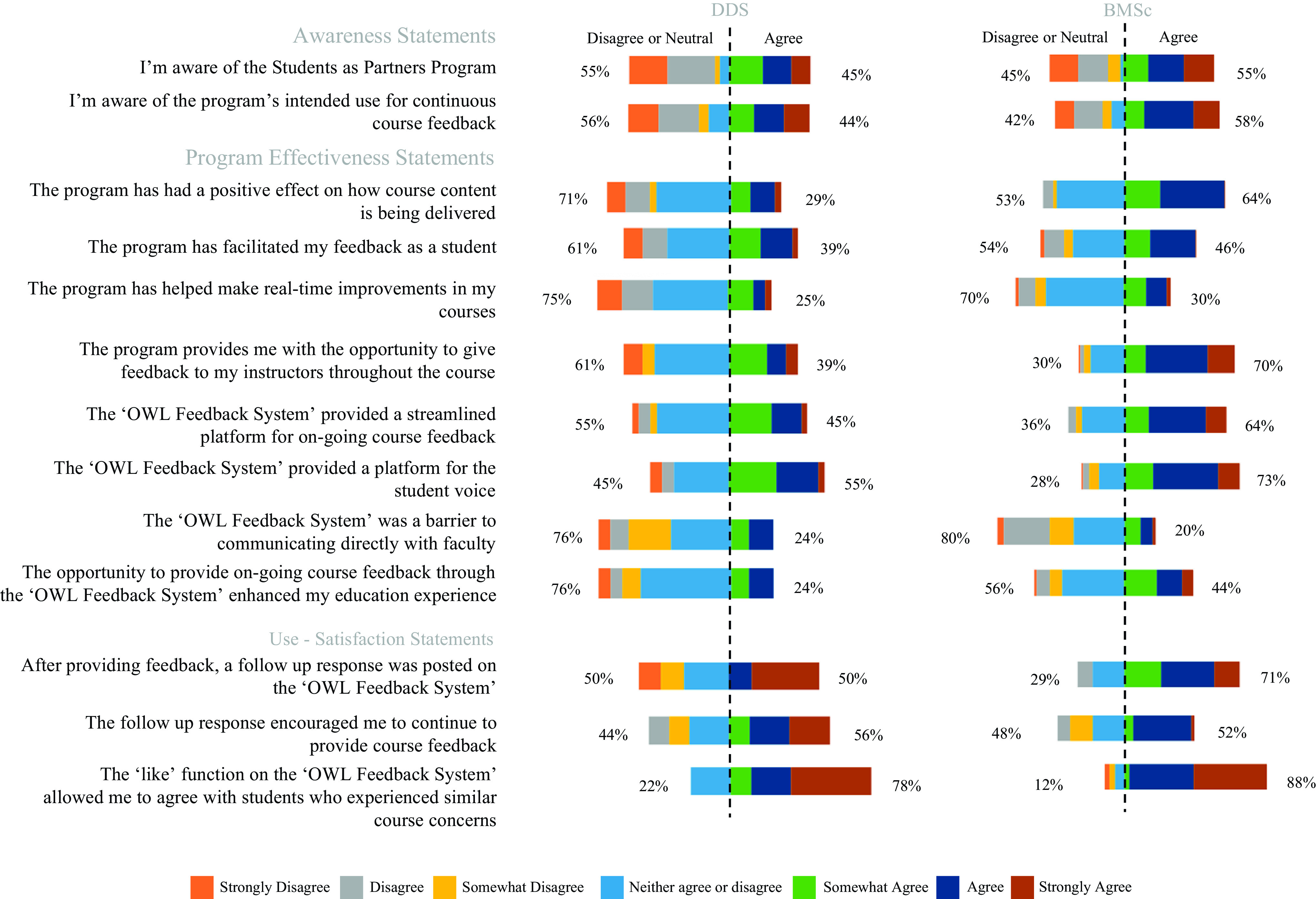
Percentage of DDS and BMSc students’ agreement with statements regarding their awareness and use-satisfaction and the effectiveness of the *cycle 2* Students as Partners (SaP) program. The colors represent the 7 different levels of agreement, ranging from “strongly disagree” to “strongly agree.” The length of the color represents the percentage of students within each agreement category. The dashed line indicates the point between “neutral” and “somewhat agree.” DDS: awareness *n* = 36–38; effectiveness *n* = 28 or 29; use-satisfaction *n* = 8 or 9. BMSc: awareness *n* = 121; effectiveness *n* = 79–92; use-satisfaction *n* = 36 or 37.

For our second iteration of the real-time feedback system, we surveyed the two student groups, the DDS and the BMSc students, as well as the participating faculty members. Both surveys assessed awareness, satisfaction, and effectiveness of the SaP program and asked open-ended questions on the program’s strengths and weaknesses. Compared with the first cycle, more of the DDS students (45%, *n* = 38) agreed that they were aware of the SaP program. The majority of BMSc student respondents (55%, *n* = 121) also agreed with this statement (Fig. 4).

With regard to effectiveness, 55% (*n* = 29) of DDS students agreed that the feedback system provided a platform for the student voice, whereas a larger majority of BMSc students (73%, *n* = 80) agreed with this statement. For use-satisfaction, it was reported that of the students who utilized the feedback system, 50% (*n* = 8) of DDS students agreed a that follow-up response was provided, whereas 70% (*n* = 37) of the BMSc students agreed with this statement.

### *Cycle 2*: Types of Student Feedback

Qualitative data collected from the students’ feedback posted on Padlet were analyzed first. The data presented in Table 1 summarize the main themes developed during the coding process. Students’ comments identified five themes for continuous course feedback: *1*) concerns about course communication and clarity, *2*) technology issues, *3*) positive instructor feedback, *4*) constructive instructor feedback, and *5*) overall course suggestions (Table 1). The two most prevalent themes from students’ feedback were *1*) concerns about course communication clarity (26.3% DDS and 33.7% BMSc) and *2*) overall course suggestions (26.5% DDS and 26.7% BMSc). Surprisingly, technology issues, a highly actionable item that could be addressed by the DMIs hired within SEED, was the lowest-reported theme by both DDS (5.9%) and BMSc (3.5%) students in *cycle 2* (Table 1). However, of the feedback that was posted by the DDS and BMSc students, there were other items that were immediately actionable and also suggestions that were actionable in a subsequent iteration of the course. Of the DDS comments 67% were actionable, and 49% of BMSc comments fell into this category (*n* = 34 DDS and *n* = 86 BMSc). Those items that were not actionable were also communicated back to students (Table 1).

**Table 1.** Real-time student feedback on the OWL Feedback System part of the SaP Program, cycle 2, Schulich School of Medicine & Dentistry, University of Western Ontario: DDS and BMSc students

Themes	Supporting Quotation(s) from DDS Students	No. (%) of Comments *n* = 34	Supporting Quotation(s) from BMSc Students	No. (%) of Comments *n* = 86
Concerns about course communication clarity	“Not communicating deadlines to us. Someone from our class told us we had an assignment during week 8. Why was it not in X’s schedule or announced to us?”	9 (26.3)	“Very minimal information was provided given what we are expected to do.”	29 (33.7)
Technology issues	“It would be helpful if X could upload transcripts or custom subtitles. I understand that there are automatic subtitles for some of her lectures, but the automatic subtitles do not do a great job of picking up her words correctly, leaving me feeling more confused.”	2 (5.9)	“Enable captions to make it easier to follow along.”	3 (3.5)
Positive instructor feedback	“I appreciate X running live demos during SIM lab. Seeing how a procedure is done, as opposed to just reading about it during didactic lectures, is exactly what we need to better understand the material.”	7 (20.6)	“X is an incredible prof who obviously cares a lot about her students and her work … she makes her expectations very clear and does her best to answer questions…”	21 (24.4)
Constructive instructor feedback	“Every so often, a SIM lab should be dedicated to students practicing any skills they want to improve on. The schedule is so packed that not everyone completes every project during SIM and unfortunately, they miss out on practicing that skill and will never have a chance to revisit that procedure until practical exams. Having lab sessions where each student works on the procedure they are least comfortable with will certainly benefit students.”	7 (20.6)	“I would find it more useful if the information was presented a different way, because I like the opportunity to look at things differently.”	10 (11.6)
Overall course suggestions	“… the exam questions asked for the GI section on the mid-term were more complicated and too detailed than needed for our knowledge. Testing a multitude of very specific proteins, receptors, and pathways may help research students, but it will do little to add to our toolbox as clinical dentists.”	9 (26.5)	“Preferred assignment-based evaluations, where we can apply knowledge from class to different contexts in cell physiology and experimentation … these help me learn way more than what I'd learn in studying for exams …”	23 (26.7)
Summary of comments with feedback	Immediate Action Completed	4 (11.8)	Immediate Action Completed	10 (11.7)
	Action Possible Next Year	17 (50)	Action Possible Next Year	32 (37.2)
	Not Actionable	4 (11.8)	Not Actionable	26 (30.2)
	Comments/Affirmations	9 (26.4)	Comments/Affirmations	18 (20.9)

### *Cycle 2*: Feedback System Strengths and Weaknesses

To better understand the perceived effectiveness of the feedback system, student survey participants were asked to comment on the factors that strengthened or weakened the program’s perceived success. Six subthemes from the qualitative data were identified for system strengths (Table 2). Both DDS and BMSc students valued the anonymity of the Padlet system, as this was the second-most prevalent theme from both cohorts. DDS students most valued the responsiveness of the SaP team (42.9%, *n* = 7), whereas BMSc students most valued the Padlet features on the feedback system (33.3%, *n* = 27).

**Table 2.** Open-ended responses from students regarding the effectiveness of the Feedback System part of the SaP Program, cycle 2, Schulich School of Medicine & Dentistry, University of Western Ontario: DDS and BMSc students

Theme and Subthemes	Supporting Quotation(s) from DDS Students	No. (%) of Comments *n* = 7	Supporting Quotation(s) from BMSc Students	No. (%) of Comments *n* = 27
*Feedback system strengths*				
Responsiveness of the SaP team	“OWL Responsiveness was quick and course directors were alerted about issues quickly.”	3 (42.9)	No quotations provided for subtheme	0 (0)
Padlet features on the feedback system	“Liking other posts.”	1 (14.2)	“…I think having the option to “upvote” or “like” others’ comments was effective. Things that were posted in the feedback system were later addressed in class, so that was shows some degree of effectiveness on the part of the instructors monitoring the system.”	9 (33.3)
Anonymity of the feedback system	“It was nice to be able to provide feedback about things that were not working well in the course without having to directly approach the professor.”	2 (28.6)	“The fact that we can communicate with the professors anonymously was a good idea to portray my feelings towards the course in real-time and allow professors to act upon the comments.”	7 (25.9)
Empowerment of the student voice	No quotations provided for subtheme	0 (0)	“Gives a voice to the students regarding what they think the course should improve on and what aspects of the course are good.”	6 (22.2)
Accessibility of the feedback system	“Usability, design features”	1 (14.2)	“The fact that it is already on OWL, which students already spend a great amount of time on, makes the whole system more effective as it requires less clicks to get to and requires remembering less usernames/passwords.”	3 (11.1)
Ability to view peers’ feedback	No quotations provided for subtheme	0 (0)	“Being able to see anonymous peer reviews is very helpful. It feels good to know that other people have the same disagreements/agreements about the way things are being taught and the content being taught.”	2 (7.4)

Six subthemes were identified from the qualitative data for system weaknesses (Table 3). The two most prevalent themes identified from DDS students’ feedback were *1*) did not access the feedback system (35%) and *2*) concerns regarding the clarity of the feedback system and program (25%) (*n* = 20). The two most prevalent themes from BMSc students’ feedback were *1*) lack of awareness (40%) and *2*) concerns regarding the clarity of the feedback system and program (34.3%) (*n* = 35).

**Table 3.** Open-ended responses from students regarding areas of improvement for the Feedback System part of the SaP Program, cycle 2, Schulich School of Medicine & Dentistry, University of Western Ontario: DDS and BMSc students

Theme and Subthemes	Supporting Quotation(s) from DDS Students	No. (%) of Comments *n* = 20	Supporting Quotation(s) from BMSc Students	No. (%) of Comments *n* = 35
*Feedback system weaknesses*				
Lack of awareness	“Out of the 12 students I just talked to in our group chat, only 1 of us had even heard about this, and they didn't even know what it was for. “OWL Feedback system” is what you use when your online quiz crashes and you need OWL tech support. The name tells us absolutely nothing about its function. Don’t call it Course Feedback, Class Feedback, or anything with “feedback” in it—this is just a red flag for students to delete that email along with the thousand other emails the university bombards us with.”	4 (20.0)	“I didn’t really know it existed till I saw it on OWL, so maybe a professor could mention its existence.”	14 (40.0)
Did not access the feedback system	“Haven’t used it.”	7 (35.0)	“I didn’t get a chance to use it.”	6 (17.1)
Concerns regarding the clarity of the feedback system and program	“Unsure if instructor use/integrate feedback, still too early to tell effect.”	5 (25.0)	“Although students get a voice, we don’t know if it is reaching the professor, and if the student’s critique is being taken into the consideration.”	12 (34.3)
Feedback system was only offered in select courses	“Not enough professors/course directors signed up for the program.”	4 (20.0)	No quotations provided for subtheme	0 (0)
No concerns reported	No quotations provided for subtheme	0 (0)	“As far as I’ve used it, the system works fine.”	2 (5.7)
Concerns regarding accessibility	No quotations provided for subtheme	0 (0)	“The feedback system is not organized, and it is hard to navigate. For the purpose we used it, as a trial, it was effective because there was not much activity, however, if many more classes were to implement it, and it became a mainstream method for student feedback, it would very quickly become overrun with student comments, almost like a Forum section of OWL that is not organized into sections …”	1 (7.4)

### *Cycle 2*: Faculty Perceptions

To determine faculty perceptions from *cycle 2* of the SaP program feedback system, core faculty participants were surveyed (Table 4). When asked about their awareness of the SaP program, 90% of faculty respondents agreed that they were aware, 50% agreed that they had a clear understanding of how the SaP program works, and 50% agreed that they were aware of the intended use of the SaP program (*n* = 10). With regard to implementation of feedback in their courses, 55.6% (*n* = 9) agreed that they were informed of feedback in their courses whereas 57.1% (*n* = 7) agreed that they implemented changes to the delivery of curriculum from the feedback they received.

**Table 4.** Faculty perceptions regarding the SaP Program, Schulich School of Medicine & Dentistry, cycle 2, University of Western Ontario: instructed courses in DDS and BMSc

Statement Category and Question	No. (%) in Agreement	No. of Responses
*Awareness*		
I’m aware of the Students as Partners program.	9 (90.0)	10
I have a clear understanding of how the Students as Partners program works.	5 (50.0)	10
I’m aware of the intended use of the Students as Partners program.	5 (50.0)	10
*Feedback implementation*		
I’ve been informed about the feedback provided in my courses.	5 (55.6)	9
I’ve implemented changes to the delivery of the curriculum in my courses from the feedback received.	4 (57.1)	7
The program has had a positive effect on my curriculum delivery models.	2 (28.6)	7
The program has facilitated a streamlined system for feedback.	3 (42.9)	7
I have been able to allocate my time more effectively in deciding where to focus course changes as a result of this program.	1 (12.5)	8
Student feedback through the program has identified areas for real-time improvements in my courses.	2 (28.6)	7
The program provided me with feedback to implement changes in curriculum delivery models for my future courses.	2 (28.6)	7
The program was a barrier to communicating directly with students.	1 (14.3)	7
The program supported me with technology teaching practices.	3 (50.0)	6
The program enhanced my teaching practices.	3 (42.9)	7
I plan to use the program in my future courses.	4 (50.0)	8

All data presented summarize the percentage of respondents in agreement with a statement (i.e., answered either “somewhat agree,” “agree,” or “strongly agree”).

Faculty respondents were also asked to comment on the factors that strengthened or weakened the program’s perceived success. Three themes emerged from faculty open-ended responses (Table 5). Of the comments submitted, 30% provided reasons for program effectiveness, 35% provided suggestions for the program, and 35% provided comments on overall impressions of the program, which were generally positive (*n* = 20).

**Table 5.** Free-text responses from faculty regarding the SaP Program, Schulich School of Medicine & Dentistry, University of Western Ontario, cycle 2: instructed courses in DDS and BMSc

Themes	Supporting Quotation(s) from Instructors	No. (%) of Comments *n* = 20
SaP program effectiveness	“What I really appreciated about the program was the feedback system. Once a student submits a feedback, other students can also agree with the feedback and that information is also provided. This helped me to gauge how many students share a specific opinion and feedback about the course. This would especially be helpful to prioritize and assess the weight of various feedbacks from students.”	6 (30.0)
	“Working with the Digital Media Intern.”	
	“Responding to student feedback with concrete examples of changes that were made to the course based on that feedback.”	
SaP program suggestions	“Increase exposure and awareness of the program.”	7 (35.0)
	“I am hoping that, in the future, there will be direct communication between the course instructor and the students. This will allow the course instructor an opportunity to address any issues quickly, without having to wait for the feedback to be emailed.”	
	“I struggled with the nature of the feedback received. Most were not actionable items but rather disgruntled comments, that were inappropriate at times targeted toward certain instructors.”	
SaP program impressions	“I think it will increase the sense of community in the classroom: the students who have legitimate concerns or feedback will be heard, while those who are missing the mark may actually learn what constructive feedback looks like and how easily it can be addressed when expressed appropriately. I see this program as having a cool, perhaps indirect, peer-to-peer mentoring/learning aspect as well.”	7 (35.0)
	“It is a fantastic program that can help newly starting instructors (like myself) to get feedbacks to improve pedagogical strategies!”	
	“I think if used appropriately by students, it could be helpful.”	
	“Not positive.”	

### Quantitative Data

#### Cycle 2: awareness, use-satisfaction, and effectiveness of the SaP program.

**Figure 5.**
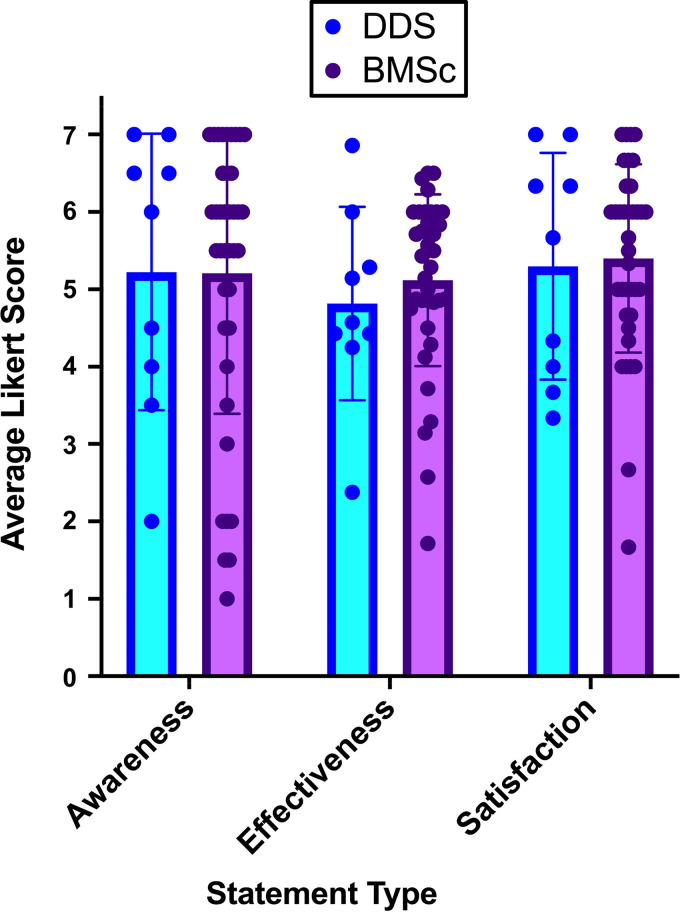
The mean Likert responses for questions about awareness, effectiveness, and satisfaction of the SaP program were compared between DDS and BMSc users (*n* = 8 DDS, *n* = 36 BMSc). The mean Likert scores for these were not significantly different from each other for students in the dentistry program compared with those in the BMSc program (*P* > 0.05, mixed-effects analysis Šídák’s multiple-comparisons test).

In the survey, data collected from students who confirmed that they did use the feedback system (users) were separated from data from students who indicated that they did not use the feedback system (nonusers). The mean Likert score for question sets about awareness were calculated for the participants who identified themselves as users for both the DDS and BMSc students (Fig. 5), and nonusers were excluded. Not surprisingly, students who were nonusers had a lower average Likert score for awareness compared with users of the system (data not shown). Similarly, the mean Likert score for question sets about effectiveness and satisfaction were calculated for those who used the feedback system. These values were then used to perform a Šídák’s multiple-comparisons test to determine whether there were differences among the average respondent score on awareness, effectiveness, or satisfaction between DDS (*n* = 8) and BMSc (*n* = 36) student users (Fig. 5).

The mean Likert score and standard deviation reported for DDS students for awareness of the SaP program was 5.22 ± 1.79, whereas for the effectiveness of the SaP program the average Likert score was 4.82 ± 1.25, and for satisfaction with the SaP program the average Likert score was 5.30 ± 1.47. Similarly, the mean Likert score reported for BMSc students’ response for awareness of the SaP program was 5.21 ± 1.82, for the effectiveness of the SaP program the Likert score was 5.12 ± 1.11, and for satisfaction with the SaP program the Likert score was 5.40 ± 1.22. There were no statistical differences in how the DDS student users responded versus the BMSc student users for awareness (*P* = 0.9999), effectiveness (*P* = 0.9082), or satisfaction (*P* = 0.9960). Thus, overall both populations of students were generally aware of the system, were satisfied with the system, and found the system relatively effective. There were no major differences between the student group populations’ perceptions.

#### Cycle 2: Feedback system usage statistics.

After creation and implementation of *cycle 2* of the feedback system, statistics on utility for both DDS and BMSc feedback system sites were collected through built-in site visit and interaction data collected on the LMS. Of the 227 DDS students who had access to the OWL feedback system, 36% (*n* = 227) visited the feedback system site and a total of 2,362 site activities were recorded. Of the 549 BMSc students who had access to the OWL feedback system, 84% (*n* = 549) visited the feedback system site and a total of 12,659 site activities were recorded. This data suggests that there was a difference in how BMSc versus DDS students accessed the SaP feedback system.

## DISCUSSION

The drastic upheaval that COVID-19 inflicted on education undoubtably made innovation a necessity. This SaP program found its place in higher education during this crisis when students and faculty came together in response to the pandemic. SaP programs, however, are not well established in many higher education institutions (16). Although students are often encouraged to take part in course evaluations, going beyond to engage students in roles as partners for curriculum design or pedagogical consultancy is a feedback model that has not been commonly utilized (16).

Accountability is a critical component to drive change and make an impact in educational institutions. Accountability can be described as both vertical and horizontal, depending on to whom one is accountable (17). Vertical accountability describes a hierarchical system that is often established when student representatives on committees become accountable to the committee or educational program rather than to the student who provides the feedback.

Alternatively, and less common, horizontal accountability is described as collaborative rather than hierarchical, driven by shared values and goals and being accountable back to the stakeholders who made the feedback (16–18). Horizontal accountability and SaP programs share the commonality of collaborative responsibilities. When partners become accountable to one another and share responsibilities to achieve a common goal, we often see the dispersal of power and a joint decision process take place during these collaborations (16, 19, 20). Furthermore, to refrain from falling into the hierarchical system of vertical accountability in a SaP program, students electing student representatives becomes critical. In the first cycle of our SaP program, we exhibited a similar electoral process for student representatives to elucidate horizontal accountability as DDS class presidents were elected by their peers (Fig. 1). However, and unexpectedly, as we reflected on the issues from *cycle 1* we noted that the accountability of student-elected representatives brought its own challenges, in that there seemed to be a lack of communication between class presidents and the dentistry class, which ultimately impacted the effectiveness of the SaP program. Thus, although we removed horizontal accountability for *cycle 2* by removing the central role of the class presidents, we argue that we better distributed the SaP program by enabling all students to provide feedback that could be immediately seen by the SEED team.

With a new cohort of dentistry students in the subsequent year of our study, we observed early on that the motivation of student representatives varied, subsequently impacting the ability for timely actions to close the feedback loop. By identifying this potential barrier of the program, we further utilized the SEED team and student DMIs to act as education representatives between students and faculty on feedback provided. In doing so, we found a more efficient method for addressing student-faculty feedback in a timely manner. The use of Padlet in *cycle 2* more efficiently gathered all students’ voices, providing the further benefit of transparency to all participants and the ability of students to agree or disagree with posted feedback (Fig. 2).

### Closing the Feedback Loop

Our findings from *cycle 2* demonstrate that the awareness, satisfaction, and effectiveness of the SaP program feedback system were not uniform but had consistent trends across DDS and BMSc students and faculty. Of the BMSc students who utilized the feedback system, most were satisfied with the response follow-up regarding their feedback (Fig. 4). This supported our objective of the study, which was to develop a feedback system that closes the feedback loop. The percentage of dentistry student participant agreement with this statement was lower (50%), although this was not surprising, as the DDS cohort used the feedback system less frequently.

An important observation with both student participant groups was that they did not agree that real-time improvements were made in their courses, with <30% agreement from both populations (Fig. 4). It has been suggested that the most important element in closing the feedback loop is the follow-up response on why some suggestions cannot be implemented (10, 21). Our findings demonstrate and support this critical component for effective practices of feedback. For use-satisfaction, it was reported that of the students who utilized the feedback system 50% and 70% of DDS and BMSc students, respectively, agreed that a response to their feedback was provided, confirming closure of the feedback loop (Fig. 4). Although most students indicated that real-time improvements were not made, the majority of participants indicated that the feedback received was sufficient to encourage further participation in our SaP program (Fig. 4). Although a larger majority of BMSc students agreed that the feedback system closed the feedback loop compared with DDS students, it was identified that Likert responses by both BMSc and DDS students who used the feedback system were not significantly different for awareness, satisfaction, and effectiveness (Fig. 5). These data suggest that our populations, although different in their stage of their academics, were similar overall in how they perceived the utility of the real-time feedback system.

When the SaP program was first launched in *cycle 1*, technological issues were a primary concern of students and faculty. Surprisingly, technology issues, a high actionable item that could be addressed by the SaP team at SEED, was the lowest-reported theme by both DDS and BMSc students in *cycle 2* (Table 1). This suggests that once *cycle 2* was implemented, and progress had been made for online teaching and learning, technology issues were no longer a primary concern for students and faculty. Instead, a prevalent theme of feedback was centered around communication and clarity for curriculum content and delivery (Table 1).

It has been reported that healthcare professional degree programs such as dentistry have shown value in obtaining student feedback for effective learning (13, 22, 23). At this point in these students’ educational journey, they are uniquely positioned with the ability to provide feedback on continuous quality improvement (CQI) initiatives, and SaP programs have proven to be excellent models for advancing CQI and educational reform (13, 22, 23). If feedback from invested students is neglected, the curricular changes that do take place may not be best suited for their learning and may inadequately engage students in a curricular pedagogy that does not fit their needs (13, 17, 22, 23). Our innovative real-time feedback system addresses these issues by supporting a model for a student voice initiative and demonstrating that not only health professional programs can benefit in obtaining student feedback, as seen from implementation of our program in the undergraduate BMSc population, a comparatively more novice learner population group.

Although we expected there to be many similarities between the two student groups of participants in the second cycle of our feedback program, we did identify some slight differences that we found interesting. The undergraduate BMSc students seemed to be more active in their use of the reporting system, with many more site visits and posts compared with the DDS students. Although there were approximately double the number of participants in the BMSc cohort, their activity was much more than double that of the DDS students. Some of this could be because the DDS program is quite time intensive; some DDS respondents indicated that they simply did not have time to use the system (Table 3). Another possibility is that the undergraduate students in the BMSc program were more engaged and/or curious about the posts of their peers. However, more compelling was that the BMSc students more strongly agreed that the program had a positive effect on course delivery, with 64% agreeing, whereas only 29% of DDS students agreed (Fig. 4). To support this finding, one theme from the BMSc student cohort was that they felt empowered when using the feedback system (Table 2). We speculate that the BMSc student population may not feel empowered when providing feedback for the end-of-term teaching/course evaluations. It may also be that students in the DDS population already feel empowered, through their position within an advanced degree program, or that their opinions are being captured in other ways, outside of our SaP program.

### Fostering Reciprocal Partnerships

The majority of faculty viewed the impact of the SaP program positively (Table 5). Comments by faculty suggested that it was helpful for instructors looking to improve pedagogical practices, increase a sense of community in the classroom, and offer a resource for student feedback when used appropriately. These comments also foster the idea of reciprocity. The “ethic of reciprocity” is a process of balanced give and take not of commodities but of contributions: perspectives, insights, forms of participation. There is equity in what is exchanged and how it is exchanged; however, those who are involved in the exchange do not get and give the same things (24). Some comments from faculty suggest that reciprocal partnerships were developed not only between students and faculty but also between students, leading to an overall improvement of the educational experience.

Alternatively, one faculty participant indicated their experience as “Not positive” (Table 4). Because of a lack of feedback in some courses, or nonactionable feedback, faculty found themselves in a position unable to make real-time improvements. For these individuals, the ethic of reciprocity was not fostered (4, 24, 25), perhaps leading to feelings of frustration or a lack of control.

### Challenges and Study Limitations

Limitations of our evaluation include its modest response rate among students, which is attributable to survey fatigue. Additionally, our results may be susceptible to response bias. Regarding our feedback system’s name, “OWL Feedback System,” some students reported confusion on the difference between our feedback system for real-time student feedback and the “Feedback.uwo.ca” tab on the LMS home page to submit their end-of-term course/instructor evaluations. Although we advertised our feedback system to students in participating courses through introduction e-mails, video tutorials, notifications on the LMS, and class president announcements, we recognize that some students overlooked these resources, subsequently impacting survey responses. Thus, survey responses for awareness statements may have been impacted.

Finally, it is important to note that because of the nature and type of qualitative study we conducted, action research, the cyclical and iterative process is one that is ongoing until an impactful program has been achieved. We recognize that additional cycles may be required to continue to evaluate the feedback system beyond measuring its ability to close the feedback loop.

### Conclusions

This study displays a framework for designing, implementing, and evaluating a real-time feedback model. Two cycles, spanning two academic terms of data analysis, have assessed the impact of a SaP program in dental and undergraduate medical science education. Our positive findings from our second cycle have encouraged us to generate a third development for further education enhancement. Students identified that they valued the ability to have a place where their viewpoints could be heard and they feel supported by their peers. Furthermore, students indicated that we offered a model that closed the feedback loop. Instructors did not view the program as a barrier to communicating with students; however, some instructors were unsure whether they would use the SaP program in future courses. Although the program may not have provided real-time improvements uniformly, our model displays how students and faculty can foster partnerships in higher education.

## DATA AVAILABILITY

Data will be made available upon reasonable request.

## GRANTS

No funding was received.

## DISCLOSURES

No conflicts of interest, financial or otherwise, are declared by the authors.

## AUTHOR CONTRIBUTIONS

N.A.S., S.M., and A.W. conceived and designed research; N.A.S. performed experiments; N.A.S., S.M., and A.W. analyzed data; N.A.S., S.M., and A.W. interpreted results of experiments; N.A.S. prepared figures; N.A.S. drafted manuscript; N.A.S., S.M., and A.W. edited and revised manuscript; N.A.S., S.M., and A.W. approved final version of manuscript.

## References

[1] Richardson JT. Instruments for obtaining student feedback: a review of the literature. Assess Eval High Educ 30: 387–415, 2005. doi:10.1080/02602930500099193.

[2] Adams S, Bekker S, Fan Y, Gordon T, Shepherd LJ, Slavich E, Waters D. Gender bias in student evaluations of teaching: ‘Punish[ing] those who fail to do their gender right’. High Educ 83: 787–807, 2022. doi:10.1007/s10734-021-00704-9.

[3] MacNell L, Driscoll A, Hunt AN. What’s in a name: exposing gender bias in student ratings of teaching. Innov High Educ 40: 291–303, 2015. doi:10.1007/s10755-014-9313-4.

[4] Mercer-Mapstone L, Dvorakova SL, Matthews KE, Abbot S, Cheng B, Felten P, Knorr K, Marquis E, Shammas R, Swaim K. A systematic literature review of students as partners in higher education. Int J Stud Partners 1: 1, 2017. doi:10.15173/ijsap.v1i1.3119.

[5] Cook-Sather A, Bovill C, Felten P. Engaging Students as Partners in Learning and Teaching: a Guide for Faculty. San Francisco, CA: Jossey-Bass, 2014.

[6] Cook-Sather A. Authorizing students’ perspectives: toward trust, dialogue, and change in education. Educ Res 31: 3–14, 2002. doi:10.3102/0013189X031004003.

[7] Mitra DL. Amplifying student voice. Educ Leadersh 66: 20–25, 2008.

[8] Subramanian J, Anderson VR, Morgaine KC, Thomson WM. The importance of “student voice” in dental education. Eur J Dent Educ 17: e136–e141, 2013. doi:10.1111/j.1600-0579.2012.00773.x. 23279401

[9] Green W. Engaging “Students as Partners” in global learning: some possibilities and provocations. J Stud Int Educ 23: 10–29, 2019. doi:10.1177/1028315318814266.

[10] Watson S. Closing the feedback loop: ensuring effective action from student feedback. Tert Educ Manage 9: 145–157, 2003. doi:10.1080/13583883.2003.9967099.

[11] Nasser F, Fresko B. Faculty views of student evaluation of college teaching. Assess Eval High Educ 27: 187–198, 2002. doi:10.1080/02602930220128751.

[12] Tschirhart C, Pratt-Adams SD. Closing the loop: an evaluation of student-led module feedback at one UK higher education institution. Int J Stud Partners 3: 78–90, 2019. doi:10.15173/ijsap.v3i2.3554.

[13] Subramanian J, Anderson VR, Morgaine KC, Thomson WM. Improving the quality of educational strategies in postgraduate dental education using student and graduate feedback: findings from a qualitative study in New Zealand. Eur J Dent Educ 17: e151–e158, 2013. doi:10.1111/eje.12006. 23279403

[14] Kemmis S, McTaggart R. Participatory action research. In: Handbook of Qualitative Research, edited by Denzin NK, Lincoln YS. Thousand Oaks, CA: Sage, 2000, p. 567–607.

[15] Sullivan GM, Artino AR. Analyzing and interpreting data from Likert-type scales. J Grad Med Educ 5: 541–542, 2013. doi:10.4300/JGME-5-4-18. 24454995 PMC3886444

[16] Healey M, Flint A, Harrington K. Engagement through Partnership: Students as Partners in Learning and Teaching in Higher Education (Online). 2014. https://www.heacademy.ac.uk/engagement-through-partnership-students-partners-learning-and-teaching-higher-education.

[17] Bilodeau PA, Liu XM, Cummings BA. Partnered educational governance: rethinking student agency in undergraduate medical education. Acad Med 94: 1443–1447, 2019. doi:10.1097/ACM.0000000000002777. 31045600

[18] Schillemans T. Does horizontal accountability work? Evaluating potential remedies for the accountability deficit of agencies. Adm Soc 43: 387–416, 2011. doi:10.1177/0095399711412931.

[19] Little S, Sharp H, Hayward M, Gannon-Leary P, O’Neil P, Williams J. Collaborating for staff-student partnerships: experiences and observations. In: Staff-Student Partnerships in Higher Education. London, UK: Continuum, 2011, p. 215–225.

[20] Williamson M. Guidance on the Development and Implementation of a Student Partnership Agreement in Universities. 2013. http://www.sparqs.ac.uk/upfiles/Student/Partnership/Agreement/Guidance-final-version.pdf.

[21] Whelehan DF. Students as Partners: a model to promote student engagement in post-COVID-19 teaching and learning. AISHE J 12: 1–10, 2020.

[22] Cardall WR, Rowan RC, Bay C. Dental education from the students’ perspective: curriculum and climate. J Dent Educ 72: 600–609, 2008. doi:10.1002/j.0022-0337.2008.72.5.tb04525.x. 18451084

[23] Scott KW, Callahan DG, Chen JJ, Lynn MH, Cote DJ, Morenz A, Fisher J, Antoine VL, Lemoine ER, Bakshi SK, Stuart J, Hundert EM, Chang BS, Gooding H. Fostering student-faculty partnerships for continuous curricular improvement in undergraduate medical education. Acad Med 94: 996–1001, 2019. doi:10.1097/ACM.0000000000002726. 30920449

[24] Cook-Sather A, Felten P. Ethics of academic leadership: guiding learning and teaching. In: Cosmopolitan Perspectives on Academic Leadership in Higher Education, edited by Su F, Wood M. London, UK: Bloomsbury, 2017, p. 175–191.

[25] Fraser SP, Bosanquet AM. The curriculum? That’s just a unit outline, isn’t it? Stud High Educ 31: 269–284, 2006. doi:10.1080/03075070600680521.

[26] Braun V, Clarke V. Using thematic analysis in psychology. Qual Res Psychol 3: 77–101, 2006. doi:10.1191/1478088706qp063oa.

